# Antifungal and proteolytic activities of endophytic fungi isolated from *Piper hispidum* Sw

**DOI:** 10.1590/S1517-838246220131042

**Published:** 2015-06-01

**Authors:** Ravely Casarotti Orlandelli, Tiago Tognolli de Almeida, Raiani Nascimento Alberto, Julio Cesar Polonio, João Lúcio Azevedo, João Alencar Pamphile

**Affiliations:** Universidade Estadual de Maringá, Laboratório de Biotecnologia Microbiana, Departamento de Biotecnologia, Genética e Biologia Celular, Universidade Estadual de Maringá, PR, Brasil, Laboratório de Biotecnologia Microbiana, Departamento de Biotecnologia, Genética e Biologia Celular, Universidade Estadual de Maringá, PR, Brazil.

**Keywords:** antagonism, competitive interaction, dual culture, cup plate, protease

## Abstract

Endophytes are being considered for use in biological control, and the enzymes they secrete might facilitate their initial colonization of internal plant tissues and direct interactions with microbial pathogens. Microbial proteases are also biotechnologically important products employed in bioremediation processes, cosmetics, and the pharmaceutical, photographic and food industries. In the present study, we evaluated antagonism and competitive interactions between 98 fungal endophytes and *Alternaria alternata*, *Colletotrichum* sp., *Phyllosticta citricarpa* and *Moniliophthora perniciosa*. We also examined the proteolytic activities of endophytes grown in liquid medium and conducted cup plate assays. The results showed that certain strains in the assemblage of *P. hispidum* endophytes are important sources of antifungal properties, primarily *Lasiodiplodia theobromae* JF766989, which reduced phytopathogen growth by approximately 54 to 65%. We detected 28 endophytes producing enzymatic halos of up to 16.40 mm in diameter. The results obtained in the present study highlight the proteolytic activity of the endophytes *Phoma herbarum* JF766995 and *Schizophyllum commune* JF766994, which presented the highest enzymatic halo diameters under at least one culture condition tested. The increased activities of certain isolates in the presence of rice or soy flour as a substrate (with halos up to 17.67 mm in diameter) suggests that these endophytes have the potential to produce enzymes using agricultural wastes.

## Introduction

Biological control through microorganisms that inhibit or antagonize plant pathogens and pests reduces or eliminates the use of chemical products. Fungal endophytes are effective antagonists (Azevedo *et al.*, 2000) and constitute a taxonomically and metabolically diverse group of organisms that colonize internal plant tissues without causing apparent harm to the host plant (Wilson *et al.*, 1991). Indeed, endophyte-mediated biological control has been investigated both *in vivo* and *in vitro* through screening experiments to verify the activity of endophytes against phytopathogens and pests (Andreote *et al.*, 2009; Badalyan *et al.*, 2002; Campanile *et al.*, 2007; Flores *et al.*, 2013; Mejía *et al.*, 2008; Rocha *et al.*, 2009; Rubini *et al.*, 2005; Sánchez *et al.*, 2007; Specian *et al.*, 2012).

Endophytic and phytopathogenic fungi compete and interact within the same ecological niche through the action of hydrolytic enzymes such as proteases and chitinases, which degrade the hyphal cell walls of pathogenic microorganisms (Almeida *et al.*, 2007; Guthrie and Castle, 2006; Sánchez *et al.*, 2007). This enzymatic activity is closely associated with the fungus-host specificity: the fungal strains of a given species isolated from the same host plant are remarkably homogeneous with respect to enzymatic production (Leuchtmann *et al.*, 1992; Petrini *et al.*, 1992). To facilitate the entry of endophytes into host tissues through natural or artificial openings, hydrolytic enzymes including pectinases, cellulases and lipases are secreted (Polizeli *et al.*, 1991).

Proteases or proteolytic enzymes have commercial importance (Rao *et al.*, 1998), as these enzymes are used in bioremediation and waste treatment, detergents, cosmetics and leather manufacture, silk degumming, animal cell culture, contact lens cleaning, therapy and diagnosis and the pharmaceutical, photographic and food industries. In addition, proteases are considered as insecticidal agents because these enzymes are required for the complete digestion of complex insect cuticles (Anwar and Saleemuddin, 1998; Gupta *et al.*, 2002; Harrison and Bonning, 2010; Hasan *et al.*, 2013; Kumar and Takagi, 1999; Murthy and Naidu, 2010; Nielsen and Oxenboll, 1998).

The medicinal plant *Piper hispidum* Sw. (Piperaceae), commonly known as "bayuyo" (Cuba), "cordoncillo" (Mexico), "jaborandi" or "falso-jaborandi" (Brazil), harbors a diverse endophytic fungal community (Orlandelli *et al.*, 2012a), including fungi presenting activity against human pathogenic bacteria (Orlandelli *et al.*, 2012b). Considering the shortage of information concerning the antifungal and enzymatic activities of the endophytes from this plant, the aim of the present study was to evaluate the antagonism and competitive interactions between endophytic and phytopathogenic fungi in dual culture experiments and to detect the proteolytic activity of these endophytes using a cup plate assay and different growth substrates.

## Materials and Methods

### Endophytic and pathogenic fungi

A total of 98 endophytic fungi were isolated from the leaves of *P. hispidum* plants located in a forest remnant in southern Brazil (Orlandelli *et al.*, 2012a) and belong to the fungal culture collection of the Laboratório de Biotecnologia Microbiana, Universidade Estadual de Maringá, Paraná, Brazil. These fungal strains were molecularly identified as *Alternaria* sp., *Bipolaris* sp., *Colletotrichum* sp., *Colletotrichum gloeosporioides*, *Phyllosticta capitalensis*, *Lasiodiplodia theobromae*, *Marasmius cladophyllus*, *Phlebia* sp., *Phoma herbarum*, *Diaporthe* sp., *Schizophyllum commune* and one isolate from the order Diaporthales. Molecular identification was based on sequencing of the ITS1-5.8S-ITS2 region of rDNA (GenBank accession numbers JF766988 to JF767008).

The plant pathogenic fungi *Alternaria alternata*, *Colletotrichum* sp., *Phyllosticta citricarpa* and *Moniliophthora perniciosa* were obtained from the Laboratório João Lúcio Azevedo, ESALQ, Universidade de São Paulo, Brazil.

For the experiments, all fungi were previously grown in Petri dishes containing potato dextrose agar (PDA) medium (Smith and Onions, 1983) at 28 °C under biochemical oxygen demand (BOD) for seven days.

### *In vitro* antagonism and competitive interactions between endophytic and phytopathogenic fungi in dual culture

A modified version of the dual culture method of Campanile *et al.* (2007) was used. Briefly, 6-mm endophyte and phytopathogen plugs were combined in triplicate and inoculated onto PDA dishes, with a 4-cm distance between each plug. Filter paper plugs inoculated with 10 μL of fungicide Derosal plus® (with a 10^−1^ dilution of methyl benzimidazol-2-ylcabamato + tetramethylthiuram disulfide) or fungicide Tiofanil® (with a 200 mg/mL dilution of chlorothalonil + thiophanate-methyl) were used as positive controls, and autoclaved distilled water was used as a negative control.

The antagonism index (AI) was calculated as previously described (Campanile *et al.*, 2007) using the following formula: AI = (RM - rm)/RM × 100, where rm represents the ray of the colony toward the antagonist, and RM represents the average of the three rays of the colony in the other directions. The competitive interaction (CI) between endophytes and phytopathogens was determined according to the Badalyan rating scale (Badalyan *et al.*, 2002), which considers three main types of interactions (A, B and C) and four interaction sub-types (C_A1_, C_B1_, C_A2_ and C_B2_). Types A and B represented deadlock (mutual inhibition) at mycelial contact (A) or at a distance (B), whereas type C was replacement or overgrowth without initial deadlock. The intermediate interaction subtypes scored consisted of partial (C_A1_) or complete (C_A2_) replacement after initial deadlock with mycelial contact and partial (C_B1_) or complete (C_B2_) replacement after initial deadlock at a distance.

### Conditions for protease production and cup plate assay

The endophytic fungi were grown as previously described (Sena *et al.*, 2006) in liquid inducer medium (IM) containing powdered skim milk (Nestlé®) as the inducer substrate to stimulate protease secretion. The cultivation conditions were adapted from Sena *et al.* (2006), and the endophytes were also grown in IM containing two different substrates (carbon sources): rice or soy flour (5 g/L). Liquid medium incubated without fungal inoculation was used as a negative control. The cultures were incubated under stationary conditions (BOD at 28 °C for 10 days). Subsequently, the liquid medium was filtered using sterile gauze to separate the fungal mycelia.

For the cup plate assay, the filtered media were inoculated (50 μL) onto Petri dishes (9 cm) containing gelatin milk agar medium (Sena *et al.*, 2006) with the surface perforated with cup plates (6-mm diameter). A commercial protease from *Aspergillus oryzae* (Sigma®) (≥ 500 U/g) was used as a positive control.

The experiment was performed in triplicate, and the dishes were incubated under BOD at 28 °C for 24 h. The enzymatic activity was evaluated as the presence of clear halos on an opalescent background and measured in millimeters (Dingle *et al.*, 1953).

### Statistical analyses

All experiments were performed using a completely randomized design (CRD) and analyzed by ANOVA (analysis of variance). The mean values were compared using the Scott-Knott test (p < 0.05) in the statistical program SISVAR 4.3 (Ferreira, 1999).

## Results and Discussion

### Evaluation of *in vitro* antagonism (AI) and competitive interaction (CI) between endophytic fungi and phytopathogens

**Figure 1:**
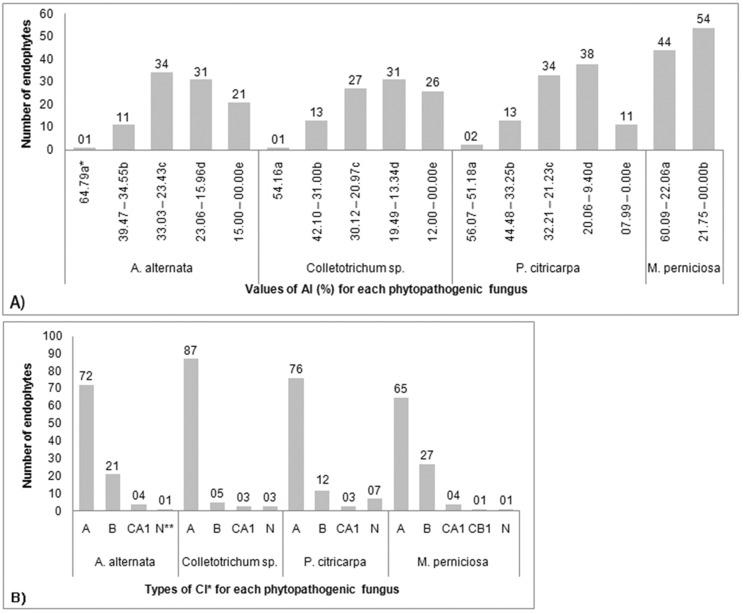
Antagonism index (AI) and competitive interaction (CI) between 98 *P. hispidum* endophytic fungi and phytopathogenic fungi in dual culture. A) AI indicates the reduction (%) in phytopathogen mycelial growth. *Means of triplicates. Different letters indicate that the AI intervals are significantly different according to the Scott-Knott test (p < 0.05). B) *Badalyan rating scale (Badalyan *et al.*, 2002): A = deadlock with mycelial contact; B = deadlock at a distance; C_A1_ = partial replacement after initial deadlock with contact; C_B1_ = partial replacement after initial deadlock at a distance. **N = no competitive interaction was observed (absence of endophyte antagonism).

The dual culture method has been broadly applied in antagonism studies because this analysis facilitates the *in vitro* screening of agents that can be used for biological control (Faria *et al.*, 2002; Mariano, 1993). In the present study, ANOVA showed differences among the *in vitro* antagonistic actions, as varying degrees of phytopathogen mycelial growth inhibition were observed. The results obtained after screening all 98 *P. hispidum* endophytes are shown in Figure 1-A, and the types of CI observed between the endophytes and *A. alternaria*, *Colletotrichum* sp., *P. citricarpa* and *M. perniciosa* are shown in Figure 1-B. More details regarding AI and CI between the 21 molecularly identified endophytes tested and phytopathogens are shown in Table 1.

**Table 1:** Antagonism index (AI) and competitive interaction (CI) between the 21 molecularly identified endophytic fungi tested and five phytopathogenic fungi in dual culture.

Endophytic fungi/controls	Phytopathogenic fungi							
	
	*A. alternata*		*Colletotrichum* sp.		*P. citricarpa*		*M. perniciosa*	
				
	AI*	CI**	AI	CI	AI	CI	AI	CI
*L. theobromae* JF766989	64.79^a^	C_A1_	54.16^a^	C_A1_	56.07^a^	C_A1_	60.09^a^	C_B1_
Diaporthales isolate JF767007	38.69^b^	A	42.10^b^	A	51.18^a^	A	37.22^a^	A
*Diaporthe* sp. JF766998	37.64^b^	A	31.00^b^	A	36.89^b^	A	27.03^a^	A
*Diaporthe* sp. JF767000	35.33^b^	A	26.90^c^	A	30.44^c^	A	34.11^a^	A
*P. herbarum* JF766995	33.03^c^	A	21.46^c^	A	28.93^c^	B	31.56^a^	A
*Bipolaris* sp. JF767007	32.33^c^	A	00.00^e^	N***	25.58^c^	A	22.29^a^	B
*Phlebia* sp. JF766997	31.40^c^	A	27.67^c^	A	27.65^c^	A	31.36^a^	A
*Bipolaris* sp. JF767001	30.49^c^	A	25.25^c^	A	24.84^c^	C_A1_	27.01^a^	A
*Colletotrichum* sp. JF766996	30.00^c^	A	11.00^e^	A	23.41^c^	A	31.91^a^	A
*C. gloesporioides* JF767002	26.65^c^	A	21.78^c^	A	29.08^c^	B	16.12^b^	A
*Bipolaris* sp. JF766993	25.28^c^	B	18.49^d^	A	22.25^c^	A	24.54^a^	A
*M. cladophyllus* JF767003	22.30^d^	A	25.00^c^	A	37.00^b^	C_A1_	08.98^b^	A
*Bipolaris* sp. JF766992	21.49^d^	A	32.36^b^	A	28.91^c^	A	32.23^a^	A
*Alternaria* sp. JF766991	20.26^d^	A	25.87^c^	A	24.05^c^	A	28.14^a^	A
*Alternaria* sp. JF766990	19.63^d^	A	25.70^c^	A	19.58^d^	A	29.43^a^	B
*Colletotrichum* sp. JF767006	17.50^d^	A	13.60^d^	A	17.42^d^	A	15.85^b^	A
*Bipolaris* sp. JF767005	13.68^e^	A	16.40^d^	A	22.01^c^	A	25.16^a^	A
*S. commune* JF766994	13.19^e^	A	17.74^d^	C_A1_	11.43^d^	A	08.88^b^	C_A1_
*Colletotrichum* sp. JF767004	12.70^e^	A	28.18^c^	A	36.69^b^	A	17.13^b^	A
*Colletotrichum* sp. JF766999	09.00^e^	A	14.28^d^	A	10.14^d^	A	21.13^b^	A
*P. capitalensis* JF766988	04.07^e^	B	11.56^e^	A	10.90^d^	B	18.18^b^	B
Fungicide Derosal Plus® ^(C+1)^	12.16^e^	-	23.00^c^	-	05.18^e^	-	23.01^a^	-
Fungicide Tiofanil® ^(C+2)^	12.16^e^	-	04.00^e^	-	03.63^e^	-	12.72^b^	-
Distilled water ^(C−)^	00.00^e^	-	00.00^e^	-	00.00^e^	-	00.00^e^	-

* Means of triplicates. The mean values followed by different letters indicate that the AI intervals are significantly different according to the Scott-Knott test (p < 0.05).

** Badalyan rating scale (Badalyan *et al.*, 2002): A = deadlock with mycelial contact; B = deadlock at a distance; C_A1_ = partial replacement after initial deadlock with contact; C_B1_ = partial replacement after initial deadlock at a distance.

*** N = no competitive interaction (absence of endophyte antagonism).

^(C+1)^Positive control (10^−1^ in distilled water); ^(C+2)^positive control (200 mg/mL in distilled water); ^(C−)^negative control.

The AI values obtained for the best antagonist (*L. theobromae* JF766989) varied between 54.16 and 64.79%, and these results were higher than those obtained in a previous study (Campanile *et al.*, 2007), where the best result for antagonism was 28.5%. Badalyan *et al.* (2002) observed that most of xylotrophic mushrooms and cereal phytopathogens present subtypes of the type C interaction. According to the scale proposed by the same authors, interaction types A and B indicate a deadlock or mutual inhibition in which neither organism overgrows in the presence of the other; in contrast, type C and associated subtype interactions indicate a replacement involving the inhibition of one organism. *L. theobromae* JF766989 partially overgrew (interactions C_A1_ and C_B1_) in the presence of all phytopathogens; however, most of the 98 *P. hispidum* endophytes presented deadlock interactions with mycelial contact (A).

Although these results suggest *L. theobromae* JF766989 as an antagonist of phytopathogenic fungi, most of the endophytes tested were more effective than fungicides for reducing the growth of the phytopathogens.

### Evaluation of the proteolytic activity of endophytic fungi

Screening for new producers of novel and industrially useful enzymes is of great interest for biotechnology research (Kumar and Takagi, 1999). Proteases are physiologically necessary and have been isolated from a wide diversity of sources, such as plants, animals, and microorganisms (Rao *et al.*, 1998). Microbial proteases have several characteristics necessary for biotechnological application and represent a large portion of the total worldwide sale of enzymes, with low production costs compared with animal or plant proteases. Moreover, microorganisms are preferred as a source of proteases due to their rapid growth, limited space requirements for cultivation and ease of genetic manipulation to generate new enzymes with desirable properties (Najafi *et al.*, 2005; Said and Pietro, 2002).

**Figure 2:**
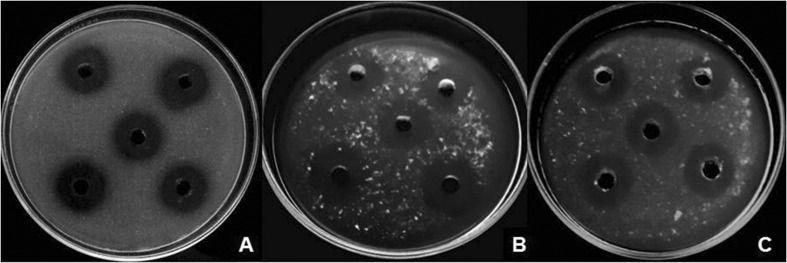
Cup plate assay: A) *Schizophyllum commune* enzymatic halos (16.40 mm) produced after growth in inducer medium (IM); B) *S. commune* halos (15.40 mm) produced after growth in IM + rice flour; C) *Phoma herbarum* halos (17.67 mm) produced after growth in IM + soy flour.

The cup plate assay in the present study showed that 28 of the 98 endophytes (28.57%) presented proteolytic activity when grown in inducer medium. An ANOVA showed differences in the observed enzymatic halos, with means ranging from 1.33 to 16.40 mm in diameter; the highest value was observed for *S. commune* JF766994 (Fig. 2 and Table 2).

**Table 2:** Proteolytic activity of endophytic fungi grown in liquid inducer medium (IM) with and without additional substrates compared with a commercial fungal protease.

Fungi	Halos (in mm) obtained for each culture condition		
	
	IM	IM + rice flour	IM + soy flour
*S. commune* JF766994	16.40^Ba^ *	15.40^Ba^	15.00^Ba^
Endophyte G62-75	15.40^B^	-	-
Endophyte G23-54	15.00^C^	-	-
*Colletotrichum* sp. JF767004	14.47^Ca^	14.53^Ba^	15.00^Ba^
Endophyte G38-111	14.00^Ca^	14.27^Ba^	13.40^Ca^
Endophyte G42-108	10.20^Da^	14.46^Ba^	-
Endophyte G13-13	10.00^D^	-	-
*L. theobromae* JF766989	09.53^D^	-	-
Endophyte G04-24	09.47^Da^	09.87^Ca^	15.00^Ba^
Endophyte G17-101	08.27^D^	-	-
Endophyte G57-82	06.67^D^	-	-
*Alternaria* sp. JF766991	06.00^D^	-	-
Endophyte G04-04	06.00^Da^	15.00^Ba^	11.33^Da^
*P. herbarum* JF766995	05.67^Eb^	10.27^Cb^	17.67^Ba^
Endophyte G36-112	05.47^Eb^	14.20^Ba^	-
Endophyte G39-39	05.47^Ea^	10.33^Ca^	-
*Bipolaris* sp. JF767001	05.40^E^	-	-
Endophyte G07-23	05.20^E^	-	-
*Bipolaris* sp. JF767005	04.87^Ea^	10.00^Ca^	10.26^Da^
Endophyte G07-138	04.47^E^	-	-
Endophyte G01-01	03.93^F^	-	-
Endophyte G54-69	03.93^F^	-	-
Endophyte G53-83	03.67^Fb^	13.87^Ba^	05.00^Eb^
*Colletotrichum* sp. JF766999	03.60^F^	-	-
Endophyte G62-127	03.53^Fa^	07.86^Da^	-
Endophyte G11-11	03.47^F^	-	-
Endophyte G05-05	02.27^Gb^	07.27^Db^	15.00^Ba^
Endophyte G35-35	01.33^Ga^	10.00^Ca^	09.73^Da^
Positive control ^(C+)^	23.63^A^	23.63^A^	23.63^A^

* Means of triplicates. The mean values followed by upper-case letters in columns or lower-case letters in rows are not significantly different according to the Scott-Knott test (p < 0.05).

^(C+)^Positive control: commercial protease from *Aspergillus oryzae* Sigma® (≥ 500 U/g) used directly in the cup plate assay.

Approximately 28.57% of the *P. hispidum* endophytes evaluated presented proteolytic activity under the conditions assayed. In some cases, the enzyme production was significantly higher after the medium was changed. Species of the genus *Mucor* are protease producers of commercial value (Alves *et al.*, 2002), with 82% of 56 isolates belonging to 11 different species presenting proteolytic activity. Djamel *et al.* (2009) reported that only 10 (3.9%) of 253 *Penicillium* strains examined presented significant proteolytic activity, as based on the hydrolysis of milk casein (clear zones around the colony) and the mycelium colony diameter, with clear halos greater than 9 mm.

The 28 *P. hispidum* endophytic isolates that initially presented enzymatic activity were grown in the presence of rice or soy flour (Table 2). When rice flour was used as the substrate, 14 endophytes produced enzymatic halos, ranging from 7.27 to 15.40 mm, with the best values observed for *S. commune* JF766994. In addition, two unidentified isolates (G53-83 and G36-112) presented statistically superior enzymatic activity when grown on this substrate. In the presence of soy flour, positive results were obtained for 10 endophytes, with enzymatic halos ranging from 5.0 to 17.67 mm in diameter. The best result was obtained for *P. herbarum* JF766995, which presented statistically superior enzymatic activity when grown on this substrate; similar results were obtained for isolate G05-05.

Agro-industrial and other wastes can be used as substrates for fermentation, suggesting a cost-effective approach to enhance enzymatic production, as these substrates are cheap and abundant natural carbon sources (Blesson, 2009; Singh *et al.*, 2012). Singh *et al.* (2012) showed that sugarcane bagasse, wheat bran, corncob, wheat straw and, in particular, rice bran are suitable substrates for the production of amylases and xylanases from thermophilic actinobacteria. In addition, substrates such as soy, wheat and rice bran, mango and banana peel, gelatin and fish flour have been used for the production of microbial proteases (Murthy and Naidu, 2010; Paranthaman *et al.*, 2009; Souza *et al.*, 2008).

Consistent with the results of the present study, Souza *et al.* (2008) used the cup plate assay to investigate the production of enzymes from Amazonian basidiomycetes cultivated on different substrates, obtaining enzymatic halos of up to 23.80 mm in diameter. Smaller halos (up to 18.07 mm in diameter) were obtained using a medium supplemented with protein sources, and halos of up to 19.11 and 18.64 mm in diameter were obtained on soy bran and fish flour, respectively. Paranthaman *et al.* (2009) verified protease production under the solid-state fermentation of *Aspergillus niger* using different varieties of broken rice as substrates, and the results varied between 44.7 and 67.7 U/g.

## Conclusions

Endophytes constitute a novel and important new source of active substances that can be employed in different biotechnological industries. Diverse strains, even members of the same endophytic fungal species, can exhibit characteristic metabolite production with enzymatic or antifungal potential. Some positive antifungal phenotypes of endophytes might reflect competition for space or nutrients, as demonstrated through dual culture experiments. The results of the present study suggest that in the assemblage of *P. hispidum* endophytes, certain strains are important sources of antifungal properties, particularly *L. theobromae* JF766989, which reduced the growth of *A. alternaria*, *Colletotrichum* sp., *P. citricarpa* and *M. perniciosa* by approximately 54 to 65%.

Investigators in Brazil should further explore the potential to generate new enzymes from microbial sources, as this country has a continental area that includes hundreds of plant species with diverse endophytes. The results of the present study highlight the proteolytic activity of the endophytes *P. herbarum* JF766995 and *S. commune* JF766994, which presented the highest enzymatic halo diameters under at least one culture condition tested. As some isolates showed increased activity in the presence of rice or soy flour as a substrate, these endophytes have the potential to produce enzymes from agriculture wastes.
